# We still need to talk about Hemolytic Uremic Syndrome: early recognition is key!

**DOI:** 10.1590/2175-8239-JBN-2023-E003en

**Published:** 2023-04-03

**Authors:** Lilian Monteiro Pereira Palma

**Affiliations:** 1 Universidade Estadual de Campinas, Departamento de Pediatria, Campinas, SP, Brazil.; 2 LUME Nefrologia e Diálise, Campinas, SP, Brazil

In this issue of the Brazilian Journal of Nephrology, Vilardouro *et al.* in the paper entitled “Hemolytic Uremic Syndrome – 24 years’ experience of a Pediatric Nephrology Unit”^1^ share data of a quarter of a century of experience in a tertiary center in Portugal.

A year ago, I was invited to write an editorial^2^,^3^ about the experience with Atypical Hemolytic Uremic Syndrome (aHUS), also in a tertiary center in Portugal.

## Why is it so important to talk about Hemolytic Uremic Syndrome (HUS)?

**Figure 1:**
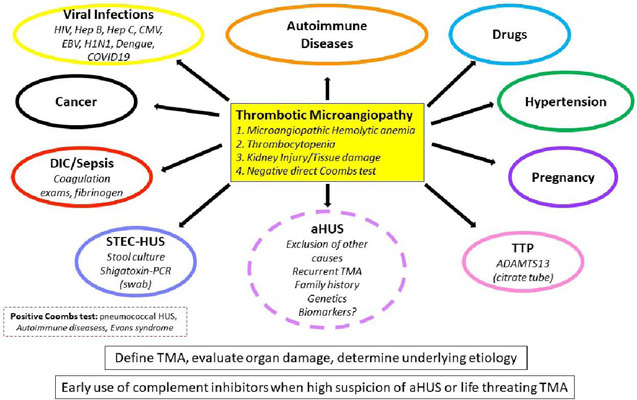
Thrombotic Microangiopathy (TMA): definition, main causes, and initial management (HIV; Human Immunodeficiency Virus; Hep B: hepatitis B; Hep C: hepatitis C; CMV: cytomegalovirus; H1N1; influenza virus, COVID19: Sars-Cov-2 virus; TTP: Thrombotic Thrombocytopenic Purpura; aHUS: atypical hemolytic uremic syndrome; STEC-HUS: shigatoxin hemolytic uremic syndrome; PCR: polymerase chain reaction; DIC: disseminated intravascular coagulation).

First, we need to define the terminology and acknowledge its changes over the years. HUS was previously used to define any manifestation of the triad of thrombocytopenia, microangiopathic hemolytic anemia, and organ injury, regardless of cause. Many centers used the term HUS to refer to shigatoxin-related HUS. The term HUS has since been replaced by Thrombotic Microangiopathy (TMA)^4^ – which is a syndrome with a devastating manifestation that can be caused by several different mechanisms. Understanding the underlying cause of TMA is the great challenge that continues to puzzle physicians today (Figure 1).

It is of utmost importance to have a checklist^5^ to:

1. Identify the presence of TMA;
2. Evaluate the degree of organ involvement;
3. Determine the underlying cause of TMA.

These three features have a huge impact on patient management and outcome.

In adults, severe deficiency of the metalloproteinase ADAMTS13 (the pathophysiology of Thrombotic Thrombocytopenic Purpura – TTP) and secondary forms of TMA are the most frequent causes. However, the secondary causes may also have an underlying complement defect and these patients could benefit from early start of complement inhibition^6^. In a recent review, we explored the role of the complement system in secondary TMA and proposed a pragmatic approach for patients with TMA^7^.

In children, infection-associated causes are the most frequent causes of TMA (shigatoxin, pneumococcal, viral) and require supportive measures. Nevertheless, the second most common cause is aHUS^8^, a consequence of the excessive formation of the membrane attack complex (MAC, C5b-9), which is a structure that resembles a pore and has the capacity to penetrate membranes leading to cell destruction by osmotic forces. C5b-9 is a product of the alternative complement pathway and is one of the first line of defenses of immunity (fundamental for the destruction of some species of bacteria and cells)^9^. The excessive formation of C5b-9 is counteracted by proteins that can return the alternative pathway back to its basal activity (also known as tick-over – an “always ready” state). Any process that leads to an imbalance in this finely tuned complement cascade may lead to the deposition of C5b-9 on endothelial cell surfaces and ultimately to the formation of microthrombi and aHUS^10^.

Becoming familiar with the most common causes of TMA may be life- and kidney-saving. Upon admission, every patient should be tested for shigatoxin (or have a stool culture prepared, which has a lower positivity), and the PLASMIC score^11^ should be applied at bedside to rule out TTP with a high degree of confidence. If available, ADAMTS13 activity should be assessed (severe deficiency is defined as activity <10% with or without a positive inhibitor).

The defects in the alternative complement pathway in aHUS may occur due to genetic mutations (gain of function of activation genes or loss of function of counter regulatory genes) or autoantibodies. Current standard of care guidelines recommend early initiation of terminal complement inhibitors^12^ to avoid death and end-stage organ damage. Eculizumab (Soliris)^13^ was approved by the North American Food and Drug Administration (FDA) for aHUS in 2011 and is a monoclonal antibody with high affinity for complement component C5, preventing C5b-9 formation. There are new complement inhibitors in the pipeline, such as ravulizumab^14^ (already approved) and others undergoing clinical trials (pegcetacoplan, crovalimab, anti-factor D, anti-factor B, lectin pathway inhibitors)^15^.

In the article in this issue of Brazilian Journal of Nephrology, Vilardouro *et al* 1 show in their series along a 24-year experience that:

1. there were 29 patients with HUS overall (average of 1 patient per year);
2. infection-related HUS was the most common cause of TMA, but only 36% of the patients had an identifiable etiology;
3. 56% of patients required kidney replacement therapy and 40% progressed to Chronic Kidney Disease and these were patients with different etiologies;
4. patients with aHUS in the pre-eculizumab era required more kidney transplants than after 2015, when eculizumab was available;
5. two-thirds of patients had sequelae at longterm follow-up;
6. both genetic testing and eculizumab were provided by the Portuguese National Health System.

Genetic testing in aHUS has a long way to go and a summit of experts is needed in my opinion. Since 2015, the pathogenicity of genetic variants has been defined^16^ but not the ideal number of genes in an aHUS genetics panel, which still varies among laboratories. Although it is very important to define transplant strategies^17^ and decide whether or not to discontinue complement inhibitors^18^,^19^, genetics is still not the gold standard diagnostic test for aHUS since positivity is variable^20^,^21^. It is important to highlight that one should not wait for the genetic test result to start treatment of suspected aHUS cases, especially if there is severe organ injury.

In conclusion, hemolytic uremic syndrome – or more specifically, thrombotic microangiopathy – is a disease with high morbidity. To prevent chronic organ damage, it is of utmost importance to address the underlying etiology quickly. There is an urgent need to engage public and private health systems^22^ in the diagnosis and protocols for early treatment according to the underlying cause.
